# An end in sight? Xrn2 and transcriptional termination by RNA polymerase II

**DOI:** 10.1080/21541264.2018.1498708

**Published:** 2018-08-02

**Authors:** Joshua D. Eaton, Steven West

**Affiliations:** The Living Systems Institute, University of Exeter, Exeter, UK

**Keywords:** Xrn2, RNA polymerase II (Pol II), transcriptional termination, 3’ end processing, cleavage and polyadenylation, RNA degradation

## Abstract

Every transcription cycle ends in termination when RNA polymerase dissociates from the DNA. Although conceptually simple, the mechanism has proven somewhat elusive in eukaryotic systems. Gene-editing and high resolution polymerase mapping now offer clarification of important steps preceding transcriptional termination by RNA polymerase II in human cells.

## Overview

The most important sequence element for transcriptional termination on protein-coding genes is the polyadenylation signal (PAS), which consists of an AAUAAA hexamer (or a variant thereof) followed by a U or GU rich element [1,2]. The former is bound by the cleavage and polyadenylation specificity factor (CPSF) complex and the latter by cleavage stimulation factors (CstF). The RNA is then cut by CPSF73 [3], whereupon the coding portion is polyadenylated and the 3ʹ product rapidly degraded. The central role of the PAS in termination provides the premise for two potential mechanisms. One is referred to as the allosteric model and proposes that a PAS-dependent conformational change elicits termination [4]. This might be in RNA polymerase II (Pol II) itself or via an exchange of its associated factors. Another is that RNA cleavage promotes termination by generating a Pol II-associated RNA, which is degraded by a 5ʹ-3ʹ exonuclease [2,5]. This torpedo model posits that the degrading exonuclease chases down Pol II and somehow signals termination.

The role of a molecular torpedo in the termination process has been controversial. RNA interference (RNAi) of the human 5ʹ-3ʹ exonuclease, Xrn2, or mutation of its budding yeast equivalent, Rat1, was originally shown to disrupt termination on transfected plasmids and endogenous genes respectively [6,7]. Although the role of Rat1 was recently re-affirmed [8], RNAi of Xrn2 did not reveal a general termination defect at the 3ʹ end of protein-coding genes [9]. However, termination was subsequently shown to be affected when a catalytically inactive version of Xrn2 is expressed in an RNAi background [10]. The inactive protein may block termination by trace amounts of Xrn2, remaining after RNAi, or prevent redundant 5ʹ-3ʹ exonucleases from accessing the nascent RNA in the Xrn2-depleted situation [11]. The role of RNA cleavage (a pre-requisite for Xrn2 function) in termination has also been debated. Electron micrographs of Pol II-transcribed genes show few examples of elongating polymerases associated with cleaved RNA [12]. Moreover, PAS cleavage has been argued as dispensable for termination in purified systems [13].

**Figure 1.**
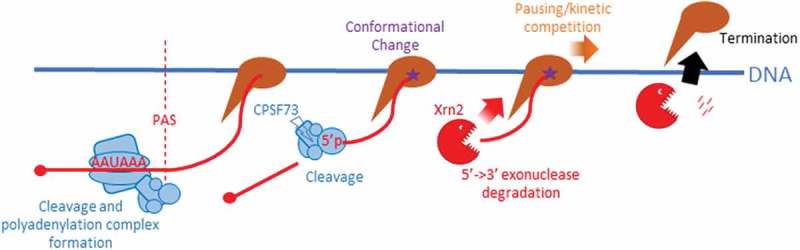
Model for the principle mechanism of Pol II termination in humans. Pol II (brown) transcribes the PAS (AAUAAA in RNA) which is bound by polyadenylation factors (blue shapes) with the RNA cleaved by CPSF73. This process likely induces a change in the elongation complex (star) rendering Pol II more susceptible to termination, which occurs through degradation of the Pol II associated product of PAS cleavage by Xrn2 (red). Xrn2-dependent termination may be augmented by Pol II pausing or arrest downstream of the PAS.

We recently addressed the role of RNA cleavage and degradation in termination by employing gene editing to bring Xrn2 or CPSF73 under inducible control [14]. Xrn2 was tagged with an auxin-inducible degron (AID) and could be eliminated within minutes whereas CPSF73 was combined with an *E.coli* DHFR degron allowing its depletion over the course of several hours. In both cases, this is much faster than the proteins can be depleted by RNAi and it revealed an unambiguous and general termination defect upon Xrn2 elimination. Loss of CPSF73 caused a strong inhibition of PAS cleavage and very extensive read-through transcription. This could not be restored by expressing its inactive derivative confirming its identity as the PAS nuclease and the importance of RNA cleavage to termination. The difference between our results and those obtained using RNAi likely reflect the speed and magnitude of depletion possible with inducible degrons. Our findings support a principal mode of termination in which PAS cleavage precedes degradation by Xrn2 and is summarised in Figure 1. These data clarify salient features of protein-coding gene termination but raise a number of interesting points for discussion.

## Gene specific effects of Xrn2 loss on termination

Mammalian native elongating transcript sequencing (mNET-seq) was used to establish the effects of Xrn2 loss on transcription [9,14]. This method provides a picture of Pol II location over the genome at single nucleotide resolution and, unlike run-on based methods, detects polymerases irrespective of their ability to transcribe. These features can be used to identify specific positions where Pol II accumulates under a variety of conditions and very precisely characterise perturbations caused by the loss of factors. We found Xrn2 depletion to induce a termination defect at the majority of protein-coding genes, but observed differences in the appearance and magnitude of effect.

In some cases, Xrn2 elimination caused enhanced signal over regions where there was already evidence of accumulated Pol II in normal cells. This may be due to Xrn2 terminating polymerases that are paused or arrested and is consistent with features that enhance Xrn2-dependent termination *in vitro* [15]. Moreover, transient transcriptome sequencing (TT-seq) identified sites of Pol II termination genome-wide and these correspond to sequences predicted to promote polymerase pausing [16]. Xrn2 loss also often results in Pol II signal beyond where transcription terminates in its presence. This may be where Pol II pausing is infrequent or because weaker pausing has been overcome due to low levels of Xrn2. As such, Xrn2 may pursue a mixture of stalled and transcribing Pol II.

While the majority of protein-coding genes show read-through upon Xrn2 loss there are cases where this is much more extensive – extending tens of kilobases further than normal. Perhaps these are examples of where Pol II does not pause and responds more acutely to Xrn2 loss or these genes may not employ alternative termination mechanisms that could act in the absence of Xrn2 (see below). Other possibilities would include slower 3ʹ end processing that would delay the production of an Xrn2 entry site or rapid Pol II elongation rates previously shown to extend transcription [10]. A more detailed analysis of primary sequence or chromatin features will be important to understand the basis of these different Xrn2 effects and whether they are variations on the same termination mechanism or represent distinct processes.

## RNA cleavage and Xrn2-independent termination?

It is important to note that, on most genes, mNET-seq and RNA-seq signals eventually drop to near-background levels even when Xrn2 is absent [14]. Analysis of individual genes suggests that this is still the case when inactive Xrn2 is expressed in the absence of Xrn2-AID suggesting that delayed termination even occurs when degradation from the 5ʹ end is blocked. In contrast, read-through is much more extensive upon loss of CPSF73 with analysis of individual genes showing little evidence of delayed termination. These differences could result from incomplete loss of Xrn2 (though the aforementioned result with inactive Xrn2 would argue against this) or a more effective depletion of CPSF73 compared to Xrn2. Also, CPSF73-DHFR takes longer to deplete than Xrn2-AID, which may have unknown secondary consequences.

Another interpretation of these data is that there is Xrn2-independent termination that still requires CPSF73, which may play a more underpinning role in the process through alternative mechanisms. Interestingly, exosome depletion stabilises some read-through RNA in the absence of Xrn2, which may derive from such processes [14]. The existence of auxiliary termination mechanisms would be reminiscent of fail-safe termination in budding yeast where, for example, compromising Rat1 leads to termination via the Nrd1 pathway [17]. Although the human equivalent of Nrd1 is not described, SETX is similar to the Nrd1-associating RNA:DNA helicase, Sen1, and is proposed to assist Xrn2 in termination [18].

More generally, does the function of CPSF73 in termination depend on its cleavage activity? Experiments using *in vitro* systems have been used to show that PAS-dependent termination can occur without RNA cleavage [13], but it is unclear whether this mechanism applies in cells where multiple processes are coordinated. We found that catalytically inactive CPSF73 does not restore transcriptional termination, when CPSF73 is lost, suggesting a strict requirement for cleavage [14]. Alternatively, inactive CPSF73 may not fully recapitulate the function of the native protein, for example in forming stable or precise interactions with other factors. Although formation of CPSF complexes is partially impaired by inactive CPSF73 this does not readily explain its complete inability to supress transcriptional read-through caused by the absence of CPSF73 [14,19]. Therefore, unless inactive CPSF73 disrupts key molecular contacts outside of its cleavage function, these data support an underpinning role of PAS cleavage in termination. It would generate an entry site for Xrn2 but, if the hypothesis that CPSF73 supports termination in the absence of Xrn2 is true, it may also promote termination by other mechanisms.

## The role of Xrn2 in termination at other Pol II gene classes

Pol II transcribes many different classes of genes, some of which use endonuclease cleavage for 3ʹ end formation. One example are so-called lnc-pri-miRNA genes that utilise microprocessor cleavage at their 3ʹ end [20]. Importantly, Xrn2 degrades the 3ʹ product of this cleavage and loss of Xrn2 causes a termination defect on lnc-pri-miRNA genes suggesting that Xrn2-dependent termination is not restricted to PAS-containing genes [14].

Other transcript classes that undergo 3ʹ end cleavage include those coding for snRNAs and replication-dependent histones (RDH). RDH genes employ a complex similar to that used for other protein-coding genes with CPSF73 as the endonuclease [21]. The 3ʹ ends of snRNAs are also formed by endonuclease cleavage, which is performed by the Ints11 subunit of the integrator complex [22]. Interestingly, Xrn2 elimination does not affect read-through at either of these gene classes. Additionally, promoter upstream transcripts (PROMPTs) are largely unaffected by Xrn2 (our unpublished findings). This is despite observations that PROMPTs are relatively rich in PAS’s with evidence that these are sometimes functional [23]. Thus, RNA cleavage does not automatically trigger Xrn2-dependent RNA degradation and termination.

These unaffected transcript classes generally derive from shorter genes which may preclude the recruitment of important protein co-factors or dictate a Pol II C-terminal domain (CTD) modification status that promotes termination differently. For example, the choice of termination pathway in budding yeast is influenced at least in part by the relative densities of Serine 5 and 2 phosphorylation (Ser5p and Ser2p) on the CTD [24]. Ser5p is higher at the beginning of the transcription cycle favouring termination by Nrd1. In contrast, Ser2p is highest at the 3ʹ end of genes and likely aids Xrn2 recruitment given evidence that Rat1 is part of a complex that is recruited to Pol II by Rtt103 – a factor with some preference towards Ser2p CTD [7].

The short nature of RDH and snRNA genes also makes them susceptible to termination via an Ars2-dependent 3ʹ end processing pathway that is not active on longer genes (showing some similarity to Nrd1 termination in budding yeast) [25]. Although the details that connect Ars2 with transcript processing are not fully elucidated, this may support different termination modes. Finally, the response of genes to Xrn2 may be specified at the promoter, which is analogous to how different transcript classes, including snRNAs, are only matured when transcription is driven by their own promoter [26]. Indeed, recent work shows the sensitivity of many *C.Elegans* protein-coding genes to Xrn2-dependent termination is determined by promoter identity [27]. Intriguingly, Xrn2 is still recruited to transcription units that are unaffected by its loss, including human RDH and snRNA genes, raising the possibility that an additional feature or factor determines its involvement in termination [10,27].

## How does Xrn2 promote termination?

The torpedo model envisages that Xrn2 chases Pol II and then signals termination by a process that is still not understood [2,5]. We have discussed how paused Pol II may constitute a frequent target for Xrn2 and data from purified systems shows that prone polymerases are more effectively terminated by 5ʹ-3ʹ exonucleases [15]. However, Xrn2 may also have to pursue elongating polymerases as modulating polymerase transcription rates affects the position of termination in cells and extended mNET-seq signal is often observed in its absence [10,14].

RNA degradation may be required to deliver other important termination factors to the polymerase. A relatively small number of proteins are directly capable of inducing Pol II termination and include Xrn2, Pcf11, Sen1 (SETX in humans) and TTF2. Pcf11 causes dissolution of stalled elongation complexes *in vitro* and Sen1 is an RNA:DNA helicase that can also terminate polymerase in purified systems [28,29]. Both of these factors likely require stalled or very slow polymerases to successfully act. This is a feature of Pol II beyond the PAS and may even be promoted by RNA degradation as shortening nascent RNA can impede elongation [16,30]. TTF2 is another strong candidate as it associates with Xrn2 [31]. RNA degradation may also locate Xrn2 in close proximity to Pol II to cause termination more directly by forming specific interactions with Pol II. It is interesting in this regard that Rat1 cannot terminate *E.coli* polymerase whereas Rat and Xrn2 promote Pol II termination in the same highly purified system [15].

In argument of an indirect role for exonucleases in cells, work performed in budding yeast showed that Pol II-associated products of PAS cleavage continued to be degraded in the presence of mutated Rat1, yet the termination defect remained [11]. In this case, degradation was taken over by Xrn1 that localises to the nucleus when Rat1 function is compromised. As Xrn1 can promote termination *in vitro* [15], its inability to do so in cells might be explained by differences in processivity or the absence of interaction partners that enable Xrn2/Rat1 to function in termination. Rat1 may also indirectly affect termination as it is required, in some cases, for the recruitment of 3ʹ end processing factors to genes, including Pcf11 [11].

In human cells, Xrn2 loss had no obvious impact on Pcf11 recruitment to genes or on PAS cleavage in general [14]. Xrn2 elimination was also sufficient to inhibit co-transcriptional degradation of the 3ʹ PAS cleavage product and cause a genome-wide termination defect. Thus, any substitution of its degradation function by Xrn1 or other nucleases is probably more limited than in budding yeast. We therefore favour a more exclusive role for Xrn2 in termination and perhaps the biggest question for future study is, still, how and whether this is direct or indirect. Further implementation of inducible protein depletion systems, like AID, will likely shed more light on the termination process by revealing the contribution(s) of various factors. Ultimately, however, this conundrum will most effectively be tackled using structural biology which has illuminated many of the other phases in the transcription cycle in such amazing detail.
